# Diagnostic evaluation of suspected aneurysmal subarachnoid hemorrhage after negative CT: risk-stratified use of lumbar puncture and CT angiography

**DOI:** 10.3389/fneur.2026.1890313

**Published:** 2026-08-24

**Authors:** Clayton Rawson, Yashieta Somani, Michael Karsy, Brandon Lucke-Wold, Mehrdad Pahlevani

**Affiliations:** 1 Cell Biology and Physiology, Brigham Young University, Provo, UT, United States; 2 Department of Neurosurgery, University of Michigan, Ann Arbor, MI, United States; 3 Department of Neurosurgery, University of Florida, Gainesville, FL, United States; 4 Department of Neurosurgery, Jamaica Hospital Medical Center, New York, NY, United States

**Keywords:** aneurysmal SAH, CT angiography, diagnostic imaging, lumbar puncture, subarachnoid hemorrhage, thunderclap headache

## Abstract

Aneurysmal subarachnoid hemorrhage (SAH) is a neurologic emergency in which delayed or missed diagnosis can result in substantial morbidity and mortality. In patients who present with clinical features concerning for SAH but have a normal or non-diagnostic non-contrast head computed tomography (CT), the optimal next diagnostic step remains controversial. Traditionally, lumbar puncture (LP) has been used to detect subarachnoid blood through cerebrospinal fluid red blood cell counts and xanthochromia. More recently, CT angiography (CTA) has emerged as an alternative because of its speed, noninvasive nature, and ability to identify treatable aneurysms. However, LP and CTA answer different clinical questions and carry distinct limitations. LP is superior for directly identifying hemorrhage, including angiography-negative SAH, but is limited by traumatic taps, interpretive variability, patient discomfort, and downstream testing prompted by equivocal results. CTA is superior for rapid vascular assessment and early procedural planning, but it does not confirm that hemorrhage occurred and may identify incidental aneurysms that lead to overdiagnosis, unnecessary consultations, invasive follow-up studies, and patient anxiety. Major guidelines are not interchangeable on this point: the 2023 American Heart Association/American Stroke Association (AHA/ASA) guideline retains a Class 1 recommendation for LP after a non-diagnostic CT in patients presenting beyond six hours or with a new neurological deficit, whereas the 2019 American College of Emergency Physicians (ACEP) clinical policy regards CTA as a reasonable alternative to LP within a shared decision-making framework. Neither document specifies which test to prefer for an individual patient, how to interpret discordant results, or how patient preference should be weighted. This review examines the diagnostic performance, downstream harms, and practical tradeoffs of LP and CTA in CT-negative suspected SAH. We argue that neither test is universally superior. Rather, LP is generally favored when the priority is ruling in or ruling out subarachnoid blood, whereas CTA is favored when rapid identification of a vascular lesion is clinically important. A risk-stratified, patient-centered approach is therefore the most appropriate strategy. We report the structured literature search identifying evidence, and we summarize guideline concordance, comparative performance of LP and CTA, and scenario-specific recommendations with graded supporting evidence in tabular form.

## 1 Introduction

SAH remains one of the most time-sensitive and high-stakes diagnoses in emergency and neurovascular medicine, given its substantial morbidity and mortality if missed or delayed. Although SAH accounts for a relatively small proportion of emergency department (ED) headache presentations, the clinical consequences of a missed diagnosis, including early rebleeding, delayed cerebral ischemia, and long-term neurological disability, are severe and often irreversible (1). Non-contrast computed tomography (NCCT) has become the cornerstone of initial evaluation due to its rapid acquisition and high sensitivity in the early hours following symptom onset. However, a critical diagnostic dilemma arises when patients with a concerning clinical presentation, such as thunderclap headache, have a normal or non-diagnostic NCCT. In these cases, clinicians must determine how best to exclude SAH while minimizing unnecessary testing and downstream harms.

Historically, LP has served as the definitive follow-up test after a negative CT, leveraging its ability to detect red blood cells (RBCs) and xanthochromia in cerebrospinal fluid (CSF). More recently, CTA has gained traction as a noninvasive alternative, driven by improvements in imaging technology and increased availability in emergency settings (2). This shift has created a tension between two fundamentally different diagnostic philosophies: detecting hemorrhage directly versus identifying its presumed vascular source. LP provides biochemical evidence of bleeding, whereas CTA provides anatomical evidence of aneurysms or vascular lesions. The increasing adoption of CTA-first strategies reflects clinicians’ preference for speed and noninvasiveness but introduces concerns about incidental findings and overdiagnosis. As a result, contemporary practice is characterized by variability rather than consensus, with significant implications for patient outcomes and healthcare utilization.

### 1.1 Literature search strategy

This article is a narrative review rather than a systematic review, and the recommendations below are not the product of a formal, protocol-driven evidence synthesis. A structured search was nonetheless performed to improve transparency and reproducibility. PubMed/MEDLINE, Embase, and the Cochrane Library were searched from 1985 through 2025 using combinations of the terms “subarachnoid hemorrhage,” “thunderclap headache,” “lumbar puncture,” “xanthochromia,” “traumatic tap,” “computed tomography angiography,” “non-contrast computed tomography,” “unruptured intracranial aneurysm,” and “clinical decision rule.” Reference lists of retrieved articles, society guideline documents, and prior systematic reviews were hand-searched for additional sources. Studies published before 2000 were included only where they are of primary historical importance in defining angiography-negative and perimesencephalic SAH.

Records were eligible if they reported diagnostic accuracy, downstream utilization, complication rates, cost, or formal guideline recommendations relevant to adults evaluated for suspected non-traumatic SAH after a negative or non-diagnostic non-contrast CT. Studies were excluded if they addressed traumatic SAH exclusively, enrolled only pediatric patients, were case reports or small case series without diagnostic accuracy data, or were published in a language other than English without an available translation. Two authors independently screened titles and abstracts and then full texts, with disagreements resolved by consensus and, where necessary, adjudication by the senior author.

Because the included literature is methodologically heterogeneous and no pooled quantitative synthesis or formal risk-of-bias assessment was undertaken, the evidence supporting each recommendation is graded descriptively using Oxford Centre for Evidence-Based Medicine levels: Level 1, systematic reviews or meta-analyses of consistent prospective diagnostic cohorts with a uniformly applied reference standard; Level 2, individual prospective diagnostic cohorts; Level 3, retrospective cohorts, non-consecutive series, or case–control studies; and Level 4, case series, expert consensus, or mechanistic reasoning. These grades are reported alongside the key studies in Table 1 and alongside each scenario-specific recommendation in Table 2. We emphasize that these grades describe the strength of the underlying evidence, not the strength of a formal recommendation, and that they should not be read as equivalent to GRADE or to the Class/Level of Evidence framework used by guideline bodies.

**Table 1:** Summary of key studies informing the diagnostic evaluation of suspected aneurysmal SAH after a negative non-contrast CT.

Study	Design (n)	Principal findings relevant to this review	Evidence level
Perry et al. (4)	Prospective multicenter cohort (3,132)	CT within 6 h of onset: sensitivity 100% for SAH in this cohort; sensitivity falls to ~85.7% beyond 6 h. Established the 6-h rule.	Level 2
Backes et al. (6)	Retrospective cohort (250)	Time-dependent decline in CT sensitivity; ~90% beyond 6 h. Supports time as the principal stratifier.	Level 3
Perry et al. (7)	Prospective multicenter derivation/refinement (2,131)	Ottawa SAH Rule: sensitivity 100% (97.2–100), specificity 15.3% (13.8–16.9). Applies only to alert, neurologically intact adults with headache peaking within 1 h.	Level 2
Perry et al. (13)	Prospective cohort (1,739)	RBC < 2,000 × 10^6^/L in the final tube with no xanthochromia excludes aneurysmal SAH: sensitivity 100% (74.7–100), specificity 91.2% (88.6–93.3). Serial tube clearance unreliable.	Level 2
Dubosh et al. (3)	Systematic review and meta-analysis	Early CT is highly sensitive for SAH; supports but does not by itself justify omission of LP.	Level 1
Carpenter et al. (27)	Systematic review and meta-analysis	Comprehensive diagnostic accuracy of history, examination, CT, and LP; LP benefits only a narrow band of pre-LP probabilities (~2–7%).	Level 1
Perry et al. (8)	Prospective validation cohort (1,153)	Ottawa SAH Rule externally validated: sensitivity 100% (94.6–100), specificity 13.6%.	Level 2
Alons et al. (21)	Meta-analysis (729)	CTA after normal examination and normal non-contrast CT: vascular abnormality in 7.4%, aneurysm in 5.4%, but a causal relationship to the headache in only 1.6%. NNS 14 overall, 61 for a non-aneurysmal abnormality.	Level 2
Perry et al. (9)	Prospective implementation study (3,672)	Implementation of the Ottawa SAH Rule and 6-h CT rule preserved sensitivity but did not reduce neuroimaging rates.	Level 2
Westerlaan et al. (16)	Systematic review and meta-analysis (50 studies)	CTA per-patient sensitivity ~98% for aneurysm detection in patients with established SAH; lower for aneurysms < 3 mm.	Level 1
Menke et al. (17)	Meta-analysis (45 studies, 3,643)	CTA sensitivity 97.2%, specificity 97.9% per patient; per-aneurysm sensitivity 95%.	Level 1
Walton et al. (5)	Systematic review and meta-analysis (37 studies)	CT < 6 h: sensitivity 98.7% (96.5–100), specificity 100%. LP with spectrophotometry: sensitivity 100%, specificity 95.2%. Visual xanthochromia: sensitivity 84.9%. Ottawa rule: sensitivity 99.5%, specificity 23.7%. 658 patients require further testing per additional case identified after a negative < 6 h CT.	Level 1
Vlak et al. (19)	Systematic review and meta-analysis	Prevalence of unruptured intracranial aneurysms ~2–3% in the general population; the denominator for incidental detection on CTA.	Level 1
Rinkel et al. (14)	Prospective cohort (113)	Outcome of angiography-negative SAH depends on hemorrhage pattern: perimesencephalic pattern benign; non-perimesencephalic pattern behaves like aneurysmal SAH.	Level 2

Evidence levels follow the Oxford Centre for Evidence-Based Medicine framework described in Section 1.1.

**Table 2:** Recommended approach by clinical scenario after a negative or non-diagnostic non-contrast CT, with the level of supporting evidence. Evidence levels describe the strength of the underlying literature, not the strength of a formal recommendation.

Clinical scenario	Recommended approach	Rationale	Evidence level
Presentation < 6 h, normal high-quality CT read by an expert reader, normal neurological examination, no anemia, reliable onset time	No further testing for SAH; consider alternative diagnoses. Discuss residual risk with the patient.	Post-test probability ~0.15%; ~658 further tests per additional case identified	Level 1
Presentation > 6 h, or non-diagnostic (rather than normal) CT, with persistent suspicion	LP with CSF RBC count and xanthochromia, preferably by spectrophotometry, at ≥ 12 h from onset	LP answers the question actually being asked; AHA/ASA Class 1	Level 1–2
Any timing, but LP contraindicated, refused, or technically not feasible (coagulopathy, anticoagulation, body habitus, patient preference)	CTA as an alternative, with explicit counseling that a negative CTA does not exclude hemorrhage	ACEP Level C; shared decision-making	Level 3
New neurological deficit, depressed level of consciousness, or hemodynamic instability	CTA first, proceeding to DSA if negative or inconclusive; do not delay for LP	Anatomical question predominates; procedural risk of LP	Level 4
CTA positive, LP negative and technically adequate	Treat as incidental aneurysm; assess rupture risk (e.g., PHASES) and refer electively	Only ~1 in 4 aneurysms in this setting is causally related	Level 2
LP positive, CTA negative	Characterize hemorrhage pattern; DSA. Perimesencephalic pattern: expectant management. Non-perimesencephalic: DSA and consider repeat vascular imaging	Outcome of angiography-negative SAH is pattern-dependent	Level 2
Traumatic tap	Interpret final-tube RBC count with xanthochromia rather than serial tube clearance; < 2,000 × 10^6^/L RBCs with no xanthochromia effectively excludes aneurysmal SAH	Prospectively derived and externally tested decision rule	Level 2
RBC-positive, xanthochromia-negative CSF	If < 12 h from onset, xanthochromia may not have developed — do not exclude SAH; if ≥ 12 h and RBC below threshold, favors traumatic tap. Confirm the detection method used.	Visual inspection has a ~ 15% false-negative rate versus spectrophotometry	Level 2
Pregnancy	LP preferred where feasible to avoid fetal radiation and iodinated contrast	Avoids ionizing radiation; no contrast exposure	Level 4
Renal impairment	LP preferred; if CTA is required, weigh contrast-associated AKI risk	CA-AKI risk concentrated in pre-existing renal dysfunction	Level 3

## 2 Clinical scenario definition

### 2.1 Patient population

The clinical scenario of interest involves patients presenting with symptoms concerning for aneurysmal SAH, most notably sudden-onset, severe (thunderclap) headache, who have undergone initial evaluation, including NCCT, but remain diagnostically uncertain. These patients often exhibit additional risk features such as exertional onset, neck stiffness, photophobia, transient loss of consciousness, or focal neurological symptoms. Despite a negative or non-diagnostic CT, clinicians may maintain a high index of suspicion based on clinical gestalt or validated decision tools. This group represents a particularly challenging population because the pretest probability of SAH is neither negligible nor definitive. Consequently, the decision to pursue further testing must balance the risk of missed diagnosis against the potential harms of additional investigations. The heterogeneity of presentation further complicates this decision, as patients vary widely in risk profiles, comorbidities, and tolerance for uncertainty.

### 2.2 Critical stratifier: time from symptom onset

Time from symptom onset to CT acquisition is a critical determinant of diagnostic accuracy and strongly influences subsequent decision-making. Modern studies have demonstrated that NCCT performed within 6 hours of headache onset can achieve very high sensitivity under optimized conditions — that is, when a current-generation multidetector scanner is used and images are interpreted by a neuroradiologist or a radiologist who routinely reads head CT (3–5). In a 2022 meta-analysis of four studies (n = 2,377), the pooled sensitivity of CT within 6 hours was 98.7% (95% CI 96.5–100) with a specificity of 100% (95% CI 99.7–100) (5). These estimates should not be generalized uncritically. They were derived in centers with subspecialty neuroradiology support, and performance degrades with older scanner generations, with less experienced readers, in profound anemia (in which extravasated blood is less hyperdense relative to CSF), and when the reported time of onset is inaccurate — a limitation that is intrinsic to a history-dependent stratifier and is rarely quantified in the source studies. However, beyond this time window, CT sensitivity declines as blood products become less conspicuous due to CSF dilution and hemoglobin degradation. This temporal decline introduces uncertainty and often necessitates additional testing to exclude hemorrhage with an acceptable level of confidence. Clinical pathways frequently dichotomize patients into those presenting within 6 hours versus those presenting later, although real-world presentations may not fit neatly into these categories. Importantly, this paper focuses on patients with persistent concern for SAH after a negative or equivocal CT, regardless of timing, while recognizing that timing remains a central factor influencing test performance and clinical judgment. Reported sensitivity beyond 6 hours falls substantially, to approximately 86–90% in individual prospective cohorts (4, 6).

### 2.3 Pretest probability and validated decision instruments

Rational test selection after a negative CT begins with an explicit estimate of pretest probability, which the preceding literature suggests is poorly calibrated when left to unstructured gestalt. The Ottawa SAH Rule is the only decision instrument for this population that has been externally validated in multiple independent cohorts. It applies only to alert, neurologically intact adults with a new atraumatic headache reaching maximal intensity within one hour, and it directs investigation if any one of six features is present: age ≥ 40 years, neck pain or stiffness, witnessed loss of consciousness, onset during exertion, thunderclap (instantly peaking) headache, or limited neck flexion on examination (7). In the refinement cohort of 2,131 patients, sensitivity was 100% (95% CI 97.2–100) with a specificity of 15.3% (95% CI 13.8–16.9) (7). Prospective validation in 1,153 patients reproduced 100% sensitivity (95% CI 94.6–100) with 13.6% specificity (8), and prospective implementation across six emergency departments did not reduce neuroimaging rates while preserving sensitivity (9). Pooled across eight studies (n = 8,114), sensitivity was 99.5% (95% CI 90.8–100) and specificity 23.7% (95% CI 15.5–34.4) (5).

Three properties of the rule constrain how it should be used. First, it is a one-way instrument: the presence of a high-risk feature mandates investigation but does not raise the probability of SAH enough to dictate any particular test. Second, its specificity is low, such that strict application directs roughly three-quarters of SAH-negative patients to further testing (5). Third, its entry criteria capture only a minority of emergency department headache presentations. The rule therefore identifies the population in which the CT-negative decision arises at all; it does not adjudicate between LP and CTA. Its role in the framework proposed here is accordingly upstream of, and not a substitute for, the test-selection decision.

For the purposes of this review we treat the CT-negative population as falling into two pragmatic strata. Patients presenting within 6 hours, with a technically adequate CT interpreted by an expert reader and no new neurological deficit, occupy a low posterior probability stratum: applying the pooled accuracy estimates above to a pre-test probability of approximately 10.8%, the post-test probability of SAH is on the order of 0.15%, and approximately 658 (95% CI 250–1,749) such patients would need to undergo further testing to identify one additional case (5). Patients presenting beyond 6 hours, those whose CT is non-diagnostic rather than normal, those with a new neurological deficit, and those in whom anemia or an uncertain onset time undermines the interpretation of the scan retain a materially higher residual probability. It is in this second stratum that the choice between LP and CTA is genuinely consequential, and the recommendations in Section 6 and Table 2 are directed principally at it.

## 3 Current guideline landscape and the either/or problem

### 3.1 Emergency medicine guidance

Emergency medicine guidance reflects the evolving diagnostic landscape by permitting either LP or CTA as acceptable next steps following a negative CT. The 2019 ACEP clinical policy on acute headache addressed this decision point directly, asking whether CTA of the head is as effective as LP for safely excluding SAH in the adult emergency department patient who remains at risk after a negative non-contrast CT. It concluded that CTA is a reasonable alternative to LP for this purpose, issued as a Level C recommendation, and explicitly recommended shared decision-making with the patient regarding the tradeoffs of the CT/LP versus CT/CTA pathways (10). The same policy concluded that a normal non-contrast CT performed within 6 hours of onset, in a patient with a normal neurological examination, is sufficient to preclude further workup for SAH. Earlier commentary anticipated this shift (11), but the ACEP policy is the formal document that operationalizes it and should be cited in its place. Two qualifications are important. A Level C recommendation reflects panel consensus and lower-quality evidence rather than a high degree of clinical certainty, and ACEP itself enumerated the potential harms of a CTA-first approach — detection of incidental aneurysms leading to unnecessary invasive procedures, higher radiation dose, and loss of the alternative diagnoses that CSF analysis can reveal, such as meningitis. The policy therefore permits CTA rather than endorsing it as diagnostically equivalent. However, these guidelines often fall short of providing clear recommendations on which modality to prefer in specific clinical scenarios. As a result, clinicians are left to interpret these recommendations in the context of individual patient factors and institutional practices. This ambiguity contributes to significant variability in care, with some centers favoring CT plus LP pathways and others adopting CT plus CTA approaches. The lack of prescriptive guidance highlights the need for more nuanced, evidence-based frameworks to support decision-making.

### 3.2 Stroke and neurovascular guidance

In contrast, stroke and neurovascular guidelines primarily focus on the management of confirmed SAH, emphasizing rapid vascular imaging and intervention once hemorrhage is identified. The most relevant such document is the 2023 AHA/ASA guideline for the management of patients with aneurysmal SAH, which supersedes the 2012 guideline and does address the CT-negative decision point, though it reaches a different conclusion from ACEP. It gives a Class 1 (Level of Evidence B-NR) recommendation that, in patients with acute onset of severe headache who present more than 6 hours from symptom onset or who have a new neurological deficit, a non-contrast head CT should be performed and, if negative for aSAH, followed by LP to diagnose or exclude aSAH (12). For patients presenting within six hours without a new neurological deficit, non-contrast CT performed on a high-quality scanner and interpreted by a board-certified neuroradiologist is considered reasonable to diagnose or exclude aSAH (Class 2a, LOE B-NR), and application of the Ottawa SAH Rule may be reasonable to identify patients at high risk (Class 2b, LOE B-NR) (12). CTA appears in that guideline principally as a tool for identifying and characterizing an aneurysmal source once hemorrhage has been established, with DSA indicated when CTA is negative or inconclusive despite a high level of concern for an aneurysmal source, rather than as a substitute for LP in the exclusion of hemorrhage (12). These guidelines provide detailed recommendations for aneurysm detection and treatment but offer limited direction for the evaluation of CT-negative patients with suspected SAH. This gap is particularly important because the decision point following a negative CT represents a critical juncture in the diagnostic pathway. Without clear guidance, clinicians must extrapolate from broader principles of SAH management, which may not fully address the nuances of this scenario. The divergence between emergency medicine and neurovascular perspectives further complicates the landscape, as each specialty prioritizes different aspects of diagnosis and management. Bridging this gap requires a more integrated approach that considers both hemorrhage detection and vascular pathology.

### 3.3 Gap statement

These two documents are frequently cited alongside one another as though interchangeable, but they are not (Table 3). They were written by different specialties, for different care settings, with different implicit reference standards and different tolerances for a missed diagnosis. ACEP addresses the undifferentiated emergency department patient with acute headache and permits a CTA-first pathway under shared decision-making; the AHA/ASA addresses the patient in whom aneurysmal SAH is the specific concern and retains LP as the Class 1 confirmatory step. A clinician who cites “the guidelines” as permitting either test is, in practice, citing ACEP; a clinician who cites them as requiring LP is citing the AHA/ASA. Table 3 sets out their points of concordance and divergence explicitly. Despite widespread acknowledgment that both LP and CTA are acceptable next steps, current guidelines do not adequately operationalize how to choose between them in specific clinical contexts. Key unanswered questions include which test to prefer based on patient characteristics, how to interpret discordant results, and how to balance diagnostic certainty against potential harms. Additionally, guidelines rarely address patient preferences or the role of shared decision-making in this process. This lack of specificity leaves clinicians to rely on individual judgment, which is shaped by training, experience, and local practice patterns. Sections 6 and 7 and Table 2 attempt to operationalize that judgment.

**Table 3:** Comparison of major guideline recommendations for the CT-negative patient with suspected aneurysmal subarachnoid hemorrhage.

Domain	2019 ACEP clinical policy on acute headache (10)	2023 AHA/ASA aneurysmal SAH guideline (12)
Target population	Undifferentiated adult ED patient with acute non-traumatic headache and non-focal neurological examination	Patient in whom aneurysmal SAH is the specific diagnostic or management concern
Authoring specialty	Emergency medicine	Stroke neurology, neurosurgery, neurocritical care, neurointervention
Non-contrast CT < 6 h from onset	Normal CT precludes further workup for SAH in patients with a normal neurological examination	Reasonable to diagnose or exclude aSAH when performed on a high-quality scanner and interpreted by a board-certified neuroradiologist (Class 2a, LOE B-NR)
Next step after negative CT (> 6 h or neurological deficit)	CTA is a reasonable alternative to LP for safely excluding SAH (Level C)	Non-contrast CT followed by LP should be performed to diagnose or exclude aSAH (Class 1, LOE B-NR)
Role of CTA	Accepted alternative to LP for exclusion of SAH	Principally for identification and characterization of an aneurysmal source once hemorrhage is established; DSA indicated if CTA is negative or inconclusive despite high concern
Clinical decision rules	Ottawa SAH Rule identified as the only validated risk-stratification tool for determining the need for neuroimaging	Application of the Ottawa SAH Rule may be reasonable to identify patients at high risk (Class 2b, LOE B-NR)
Shared decision-making	Explicitly recommended for the choice between CT/LP and CT/CTA	Not specified for this decision point
Stated harms of the CTA-first pathway	Incidental aneurysms leading to unnecessary invasive procedures; higher radiation dose; loss of alternative diagnoses obtainable from CSF	Not addressed as a distinct pathway
Practical implication	Either pathway defensible; test choice individualized	LP is the expected confirmatory step in the at-risk CT-negative patient

## 4 Diagnostic performance: what each test is best at

### 4.1 Lumbar puncture (LP)

A lumbar puncture (LP) analyzes cerebrospinal fluid (CSF) to check for signs of subarachnoid hemorrhage (SAH). Clinicians primarily rely on two CSF findings: the red blood cell (RBC) count and xanthochromia (13). Xanthochromia is a change in CSF color caused by breakdown products of blood; it usually appears after the bleeding event and can remain detectable for several days. A key advantage of LP is that it tests for blood in the CSF rather than looking for the aneurysm itself; therefore, LP can still detect SAH even when vascular imaging does not identify an aneurysm, either because the hemorrhage is angiography-negative or because the bleeding source is not visible on early imaging (14, 15). Angiography-negative SAH varies: some cases are perimesencephalic hemorrhage, which usually has a benign course and very low risk of rebleeding; other cases are non-perimesencephalic angiography-negative SAH, which tends to behave more like classic aneurysmal SAH and carries a higher risk of complications (14). For this reason, a negative aneurysm study does not automatically rule out clinically important hemorrhage, and LP plays a critical role in confirming bleeding when imaging is inconclusive. Quantitatively, CSF analysis after a negative CT performs well when spectrophotometry is used: in a pooled analysis of three studies (n = 1,235), sensitivity was 100% (95% CI 100–100) with a specificity of 95.2% (95% CI 86.0–98.5) (5). Visual inspection for xanthochromia, which remains the predominant method in North American practice, performs considerably worse, with a pooled sensitivity of 84.9% (95% CI 60.0–95.5) and a false-negative rate of 15.1% (5). This distinction is frequently elided in discussions of “LP sensitivity” and is a material source of the heterogeneity in the published estimates; the confidence intervals around the visual-inspection estimates are wide because the prevalence of SAH in the underlying CT-negative cohorts is on the order of 1–2%, and the pooled estimates therefore rest on very small numbers of true-positive events.

A major limitation of LP, however, is that traumatic taps are common and can significantly complicate interpretation. If blood enters the sample as a result of the procedure itself, RBCs may appear in the CSF even in the absence of true SAH, creating diagnostic ambiguity (13). Traditional methods, such as assessing RBC clearing across collection tubes, are unreliable, as patterns may overlap between traumatic taps and true hemorrhage. A prospectively derived decision rule offers a more defensible approach: in a cohort of 1,739 CT-negative patients, the combination of fewer than 2,000 × 10^6^/L RBCs in the final tube and no xanthochromia excluded aneurysmal SAH with a sensitivity of 100% (95% CI 74.7–100) and a specificity of 91.2% (95% CI 88.6–93.3) (13). External validation in an unselected population that was not pre-screened with CT produced a markedly lower sensitivity of 81.6% (95% CI 68.0–91.2), with a traumatic tap incidence of 24.4% (5). The rule should therefore be applied only in the CT-negative population in which it was derived, and the very wide confidence interval around its sensitivity, a consequence of only a handful of SAH events, warrants explicit acknowledgment rather than the false precision implied by a point estimate of 100%. Additionally, variability in xanthochromia detection methods across institutions further complicates interpretation and limits the generalizability of findings. LP also requires technical expertise and may be influenced by patient factors such as body habitus and anticoagulation status. Despite these limitations, LP remains uniquely capable of directly confirming or excluding hemorrhage, making it a critical component of the diagnostic pathway in selected patients.

### 4.2 CT angiography

Another approach is CT angiography (CTA), which visualizes intracranial blood vessels to detect aneurysms or other vascular lesions that could cause hemorrhage. Unlike LP, it does not directly test for subarachnoid blood but instead identifies potential sources of bleeding (16). CTA is fast, widely available, and noninvasive, making it an attractive option in time-sensitive settings such as the emergency department. Modern multidetector CTA systems provide high-resolution images that can detect aneurysms with high sensitivity, particularly for lesions larger than a few millimeters (17, 18). In a systematic review and meta-analysis of 50 studies of patients with acute SAH, per-patient sensitivity of CTA for aneurysm detection was approximately 98% with specificity approaching 100% (16); a contemporaneous meta-analysis of 45 studies (n = 3,643) reported an overall sensitivity of 97.2% and specificity of 97.9%, falling to a per-aneurysm sensitivity of 95% (17). Two caveats limit the transferability of these figures to the population considered here. Both meta-analyses enrolled patients with established SAH, in whom aneurysm prevalence is high and the pretest probability of a culprit lesion is therefore very different from that of the CT-negative patient; and sensitivity falls appreciably for aneurysms smaller than 3 mm, which are precisely the lesions most likely to be encountered incidentally. Additionally, CTA offers immediate anatomical information that can facilitate early neurosurgical planning and intervention if an aneurysm is identified.

However, CTA’s primary limitation lies in its inability to confirm that a detected aneurysm is responsible for the patient’s symptoms. Unruptured intracranial aneurysms (UIAs) are relatively common in the general population, with prevalence estimates ranging from 2 to 3% (19). As a result, incidental aneurysms may be identified in patients whose headaches are unrelated to hemorrhage, leading to potential overdiagnosis and unnecessary interventions. Without corroborating evidence of hemorrhage, such as blood on CT or xanthochromia on LP, attributing symptoms to an aneurysm can be misleading (20). The magnitude of this problem has been quantified. In a meta-analysis of 729 patients with acute headache, a normal neurological examination, and a normal non-contrast CT, CTA demonstrated a vascular abnormality in 7.4% (95% CI 5.5–9.3), of which the great majority were aneurysms (5.4%; 95% CI 3.8–7.0); however, a clear causal relationship between the headache and the CTA finding could be established in only 12 patients (1.6%). The number needed to scan was 14 to detect any abnormality, but 61 to detect an abnormality other than an aneurysm (21). In other words, for every patient in whom CTA identifies a lesion that explains the presentation, roughly three are identified in whom it does not. This is the central asymmetry between the two tests and is not adequately conveyed by comparing their sensitivities. The included studies were predominantly retrospective and heterogeneous in their indications for CTA, so the absolute yield should be regarded as an approximation. An additional emerging consideration is the role of high-resolution vessel wall magnetic resonance imaging (VW-MRI), particularly black-blood MRI sequences, in the evaluation of suspected aneurysmal SAH. Unlike CTA, which primarily identifies aneurysm presence and morphology, black-blood MRI may provide additional information regarding aneurysm wall enhancement and inflammatory changes associated with recent rupture or micro-rupture (22, 23). This distinction is clinically important because CTA can identify incidental unruptured aneurysms but may not reliably determine whether an aneurysm has recently bled, particularly in cases of small-volume or angiography-negative hemorrhage. In contrast, vessel wall enhancement on black-blood MRI has been associated with aneurysm instability and rupture status in several studies (24–26). Although LP remains the gold standard for directly detecting subarachnoid blood when CT is negative, black-blood MRI may emerge as a valuable adjunct in distinguishing culprit aneurysms from incidental lesions and in evaluating suspected micro-rupture when CTA findings are equivocal. CTA also has reduced sensitivity for very small aneurysms and may be influenced by scanner quality and reader expertise. These limitations underscore the importance of interpreting CTA findings within the broader clinical context rather than in isolation.

### 4.3 What is the truth standard?

Determining a definitive “truth standard” for SAH diagnosis is inherently challenging due to limitations in each diagnostic modality and the absence of a universally accepted gold standard. Historically, the combination of NCCT followed by LP has served as a practical reference approach, leveraging the complementary strengths of imaging and CSF analysis (27, 28). However, the increasing use of CTA has shifted this paradigm, with some clinicians favoring vascular imaging as a surrogate for hemorrhage detection. Digital subtraction angiography (DSA) remains the most definitive test for aneurysm characterization, but is invasive and not routinely used as a first-line diagnostic tool.

All diagnostic pathways are subject to verification bias, as not all patients undergo all tests, and higher-risk patients are more likely to receive a comprehensive evaluation (27). Additionally, angiography-negative SAH is well described and heterogeneous, further complicating interpretation (6, 14). Observational studies examining shifts in diagnostic strategies have not consistently demonstrated differences in short-term outcomes, but these findings are limited by confounding and incomplete follow-up (3). Consequently, no single pathway can be considered a definitive gold standard, and clinical judgment remains essential in interpreting results and guiding management. More generally, the evidence base underpinning this review is dominated by prospective diagnostic cohorts from a small number of Canadian and European academic centers, single-institution retrospective series, and meta-analyses of both. Verification bias, spectrum bias arising from enrollment at tertiary centers, and the near-universal absence of blinded, uniformly applied reference standards mean that reported sensitivities are likely to be optimistic and that the confidence intervals reported throughout this review understate the true uncertainty. Where a recommendation below rests on Level 3 or Level 4 evidence, this is stated in Table 2 rather than left implicit.

## 5 Harms, downstream consequences, and false reassurance

### 5.1 LP harms and costs

Post-dural puncture headache (PDPH) is the most common complication of LP and can significantly impact patient comfort and recovery (29). Although typically self-limited, PDPH may require additional interventions such as an epidural blood patch, increasing healthcare utilization. LP also requires procedural time and expertise, potentially prolonging ED length of stay and contributing to resource strain. In addition, patient anxiety and discomfort associated with the procedure may influence acceptance and satisfaction.

Traumatic taps represent a major source of downstream consequences, as indeterminate CSF findings often prompt additional testing, including CTA or DSA, as well as observation or repeat LP. In large cohort studies, abnormal CSF findings are far more common than confirmed aneurysmal SAH, illustrating how LP interpretation can drive substantial downstream utilization (13). This cascade effect may lead to unnecessary imaging, hospital admissions, and specialist consultations. Complication rates are not trivial in absolute terms: across cohorts reporting them, 5–10% of patients undergoing LP experienced a procedure-related complication resulting in an unplanned return to the emergency department or hospitalization, with one series reporting 13 of 245 (5.3%) such events among low-risk patients in whom no case of SAH was identified (5, 30). Because these figures derive from retrospective cohorts with variable ascertainment of follow-up, they should be interpreted as a plausible range rather than a precise estimate. While LP remains a valuable diagnostic tool, its potential to generate ambiguous results must be carefully considered in clinical decision-making.

### 5.2 CTA harms and costs

Increased use of CTA has been associated with higher detection rates of incidental unruptured intracranial aneurysms, particularly in patients undergoing evaluation for headache (19). These findings often trigger a cascade of downstream interventions, including repeat imaging, neurosurgical consultations, and invasive procedures such as DSA. While some aneurysms may warrant intervention, many small incidental lesions have a low risk of rupture and may not benefit from treatment (31). This raises concerns about overdiagnosis and overtreatment, as well as the psychological burden placed on patients.

CTA also involves exposure to iodinated contrast and ionizing radiation, both of which carry potential risks. Contrast-associated acute kidney injury (CA-AKI) is most relevant in patients with pre-existing renal dysfunction, although the absolute risk in patients with normal kidney function is relatively low (32). Radiation exposure contributes to cumulative lifetime risk, particularly in younger patients or those requiring repeated imaging (33). These considerations highlight the importance of judicious use of CTA, particularly in populations at higher risk for adverse effects.

### 5.3 Missed diagnoses in real-world practice

Missed SAH remains a critical concern across diagnostic pathways, as even low rates of misdiagnosis can have significant clinical consequences. Observational studies suggest that shifts toward CTA-based strategies have not resulted in substantial increases in short-term missed SAH rates, but these findings are limited by confounding and incomplete follow-up (3). Additionally, the definition of missed SAH varies across studies, complicating comparisons. Diagnostic drift over time may also influence outcomes, as increasing reliance on imaging may lead to decreased use of LP and potential changes in detection patterns. The balance between sensitivity and specificity remains a central challenge, as efforts to reduce missed diagnoses may increase false positives and downstream harms. Ultimately, the goal is not simply to maximize detection but to optimize patient-centered outcomes, including safety, quality of life, and resource utilization.

## 6 When one is likely superior

**Figure 1:**
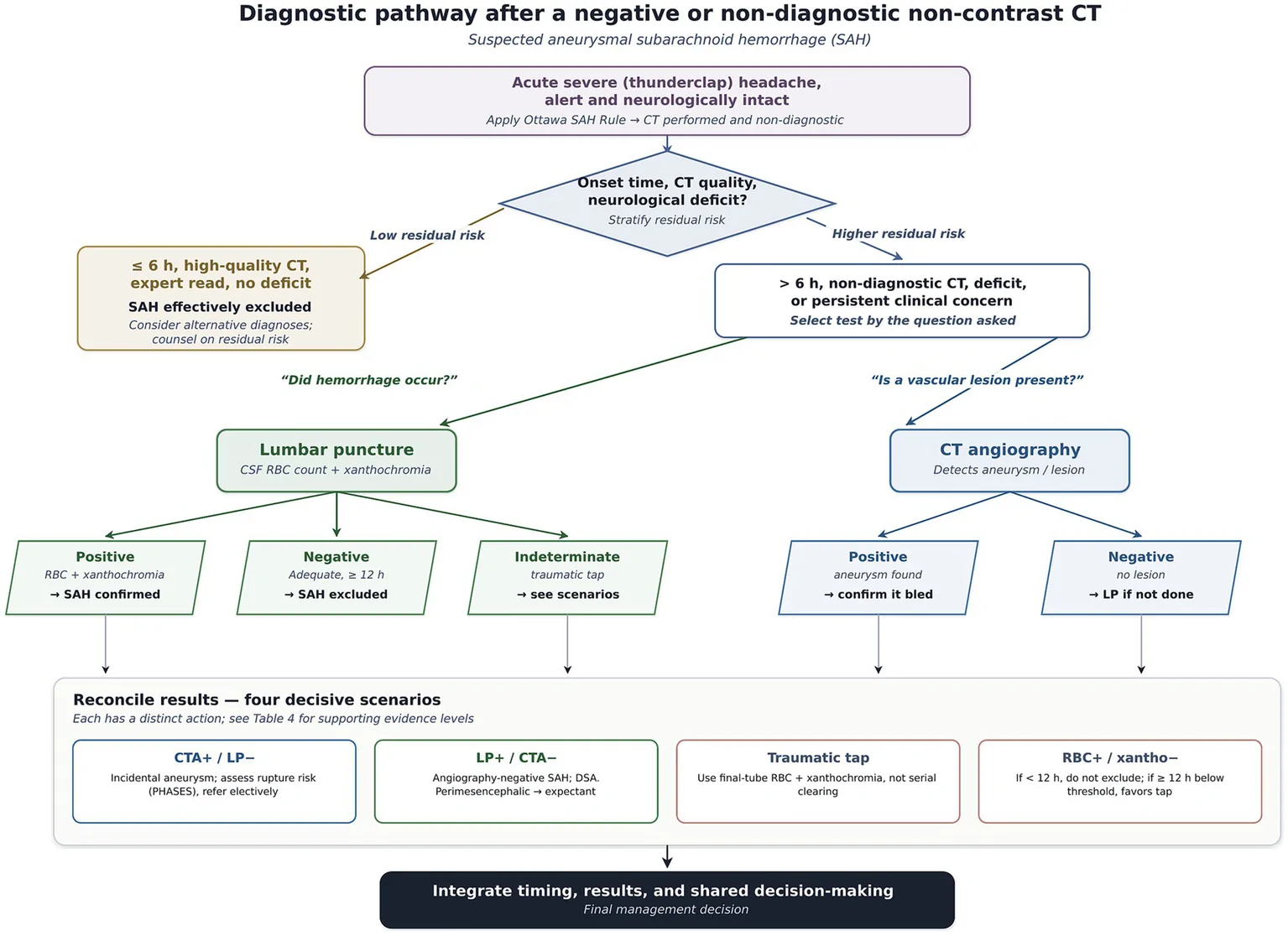
Diagnostic pathway for patients with suspected subarachnoid hemorrhage (SAH) and a negative or non-diagnostic non-contrast CT. Patients are stratified by time from symptom onset, as CT sensitivity declines beyond 6 h. Further evaluation proceeds with either LP, which detects subarachnoid blood, or CTA, which identifies vascular lesions. The figure outlines the interpretation of LP and CTA results, including indeterminate findings and discordant scenarios. Final management should integrate clinical context, diagnostic results, and shared decision-making.

A practical, risk-stratified diagnostic approach to CT-negative suspected SAH is outlined in Figure 1 and specified scenario-by-scenario, with the level of supporting evidence, in Table 2. Table 4 summarizes the head-to-head comparison of LP and CTA on which those recommendations rest. In clinical practice, the relative superiority of LP over CTA depends on the specific diagnostic question and the clinical context in which it arises. LP is generally favored when the primary concern is confirming or excluding subarachnoid hemorrhage, particularly in patients presenting more than 6 h after symptom onset or when CT sensitivity is reduced. CTA is often preferred when rapid identification of a vascular lesion is required, such as in unstable patients or when immediate neurosurgical planning is anticipated. Patient-specific factors, including renal function, pregnancy status, and tolerance for invasive procedures, also play a critical role in decision-making. Importantly, shared decision-making should be incorporated whenever feasible, as patient preferences regarding procedural invasiveness and diagnostic certainty may influence test selection. Two specific circumstances deserve emphasis because they are commonly mishandled. In the patient who presents beyond 6 hours with a normal CT and a persistently high clinical suspicion, CTA does not answer the question being asked, it cannot establish that hemorrhage occurred, and substituting it for LP converts a diagnostic question into an anatomical one. Conversely, in the patient with a new neurological deficit, a depressed level of consciousness, or coagulopathy, LP is either contraindicated or diagnostically secondary, and CTA (proceeding to DSA if inconclusive) is the appropriate first step.

**Table 4:** Head-to-head comparison of lumbar puncture and CT angiography after a negative non-contrast CT.

Characteristic	Lumbar puncture	CT angiography
Question answered	Did subarachnoid hemorrhage occur?	Is a treatable vascular lesion present?
Sensitivity (after negative CT)	100% (100–100) by spectrophotometry; 84.9% (60.0–95.5) by visual inspection (5)	97–98% per patient for aneurysm detection in cohorts with established SAH (16, 17); not a test for hemorrhage
Specificity	95.2% (86.0–98.5) by spectrophotometry; 97.6% by visual inspection (5)	97.9% for aneurysm detection (17); specificity for the cause of the presenting headache is far lower
Negative predictive value in this population	Very high; a technically adequate negative LP effectively excludes SAH	High for aneurysm, but a negative CTA does not exclude angiography-negative SAH
Positive result interpretation	Confirms blood in the CSF; requires distinction from traumatic tap	Aneurysm found; causal relationship established in only ~1 in 4 (21)
Principal failure mode	Traumatic tap (up to ~24% in unselected series); operator dependence; institutional variation in xanthochromia method	Incidental aneurysm detection; reduced sensitivity for lesions < 3 mm
Complications	Post-dural puncture headache; 5–10% unplanned return or admission in reporting cohorts (5, 30)	Iodinated contrast exposure (CA-AKI, chiefly with pre-existing renal dysfunction); ~2.5 mSv additional radiation
Resource and time burden	Procedural time and expertise; prolongs ED length of stay; laboratory turnaround for spectrophotometry	Rapid acquisition; no procedural time; requires contrast and scanner availability
Ancillary diagnostic yield	Meningitis, intracranial hypertension, other CSF-diagnosable conditions	Cerebral venous thrombosis, dissection, RCVS (NNS 61 for a non-aneurysmal abnormality) (21)
Downstream cascade	Indeterminate CSF prompts CTA, DSA, repeat LP, or observation	Incidental aneurysm prompts DSA, serial imaging, neurosurgical referral, and patient anxiety
Relative cost	Lower direct test cost; costs accrue through length of stay and complications	Higher direct test cost; costs accrue through downstream investigation of incidental findings

CA-AKI, contrast-associated acute kidney injury; DSA, digital subtraction angiography; ED, emergency department; NNS, number needed to scan; RCVS, reversible cerebral vasoconstriction syndrome.

## 7 Discordant results

Discordant results between LP and CTA present a particularly challenging clinical scenario and require careful interpretation within the broader clinical context. Four patterns account for most of these situations, and each has a different implication; they are set out with recommended actions and supporting evidence levels in Table 2. First, a positive CTA with a negative LP: given a background unruptured aneurysm prevalence of 2–3% (19) and the finding that only about one in four aneurysms detected on CTA in this setting is causally related to the presentation (21), the far more likely explanation is an incidental aneurysm. If the LP was technically adequate, performed at least 12 h after onset, and analyzed for xanthochromia, hemorrhage is effectively excluded and the aneurysm should be managed on its own merits using rupture-risk assessment such as the PHASES score (31) rather than treated as a culprit lesion. Second, a positive LP with a negative CTA: this raises the possibility of angiography-negative SAH, which is heterogeneous. A perimesencephalic pattern carries a benign course and a rebleeding risk under 1%, whereas a non-perimesencephalic pattern behaves more like aneurysmal SAH and warrants DSA and, if that is negative, consideration of repeat vascular imaging (12, 14). Third, a traumatic tap: serial tube RBC clearance is unreliable, and interpretation should instead rest on the final-tube RBC count together with xanthochromia, with fewer than 2,000 × 10^6^/L RBCs and absent xanthochromia effectively excluding aneurysmal SAH in a CT-negative patient (13). Where the result remains indeterminate, vascular imaging rather than repeat LP is generally the more informative next step. Fourth, xanthochromia-negative but RBC-positive CSF: the critical variable is the interval between symptom onset and the tap. Before roughly 12 h, xanthochromia may not yet have developed, and a negative result does not exclude hemorrhage; beyond that window, a substantial RBC count without xanthochromia favors a traumatic tap, particularly if the RBC count falls below the threshold above. The method of xanthochromia detection must also be considered, since visual inspection has a pooled false-negative rate of approximately 15% compared with essentially none for spectrophotometry (5). These scenarios highlight the limitations of each modality and the importance of integrating clinical judgment with diagnostic findings. Ultimately, management decisions should be guided by a combination of test results, clinical presentation, and risk assessment.

## 8 Future research priorities

Despite advances in diagnostic technology, significant gaps remain in the evidence base guiding the evaluation of suspected SAH. Prospective studies comparing LP and CTA using patient-centered outcomes, including miss rates and downstream harms, are needed to better inform clinical decision-making. Standardization of LP interpretation criteria, particularly regarding RBC thresholds and xanthochromia detection methods, would improve consistency and reliability. Development of integrated risk prediction tools combining clinical features, imaging timing, and diagnostic test results could further enhance accuracy and efficiency. Additionally, long-term follow-up studies are needed to assess the impact of incidental aneurysm detection and subsequent management strategies. Addressing these gaps will be essential to refining diagnostic pathways and improving patient outcomes.

## 9 Conclusion

There is no single superior diagnostic strategy for all patients with suspected SAH and a negative CT, as both LP and CTA offer distinct advantages and limitations. LP remains the most direct method for confirming or excluding subarachnoid blood, particularly when CT sensitivity is reduced or when clinical suspicion remains high. CTA, on the other hand, provides rapid, noninvasive identification of vascular lesions but carries risks related to incidental findings and downstream interventions. The two tests are not substitutes: LP answers whether hemorrhage occurred, CTA answers whether a treatable lesion is present, and the correct choice follows from which question the clinician actually needs answered. The scenario-specific recommendations in Table 2, and the guideline comparison in Table 3, are offered as a practical operationalization of that principle rather than as a substitute for the formal, prospectively validated pathway that this field still lacks.

## References

[1] van Gijn J Kerr RS Rinkel GJE. Subarachnoid haemorrhage. Lancet. (2007) 369:306–18. doi: 10.1016/S0140-6736(07)60153-6 17258671

[2] McCormack RF Hutson A. Can computed tomography angiography of the brain replace lumbar puncture in the evaluation of acute-onset headache after a negative noncontrast cranial computed tomography scan? Acad Emerg Med. (2010) 17:444–51. doi: 10.1111/j.1553-2712.2010.00694.x, 20370785

[3] Dubosh NM Bellolio MF Rabinstein AA Edlow JA. Sensitivity of early brain computed tomography to exclude aneurysmal subarachnoid hemorrhage: a systematic review and meta-analysis. Stroke. (2016) 47:750–5. doi: 10.1161/STROKEAHA.115.011386, 26797666

[4] Perry JJ Stiell IG Sivilotti MLA Bullard MJ Emond M Symington C . Sensitivity of computed tomography performed within six hours of onset of headache for diagnosis of subarachnoid haemorrhage: prospective cohort study. BMJ. (2011) 343:d4277. doi: 10.1136/bmj.d4277, 21768192 PMC3138338

[5] Walton M Hodgson R Eastwood A Harden M Storey J Hassan T . Management of patients presenting to the emergency department with sudden onset severe headache: systematic review of diagnostic accuracy studies. Emerg Med J. (2022) 39:818–25. doi: 10.1136/emermed-2021-211900, 35361627 PMC9613855

[6] Backes D Rinkel GJE Kemperman JTJH Linn FHH Vergouwen MDI. Time-dependent test characteristics of head computed tomography in patients suspected of nontraumatic subarachnoid hemorrhage. Stroke. (2012) 43:2115–9. doi: 10.1161/STROKEAHA.112.658880, 22821609

[7] Perry JJ Stiell IG Sivilotti MLA Bullard MJ Émond M Symington C . Clinical decision rules to rule out subarachnoid hemorrhage for acute headache. JAMA. (2013) 310:1248–55. doi: 10.1001/jama.2013.278018, 24065011

[8] Perry JJ Sivilotti MLA Sutherland J Hohl CM Émond M Calder LA . Validation of the Ottawa subarachnoid Hemorrhage rule in patients with acute headache. CMAJ. (2017) 189:E1379–85. doi: 10.1503/cmaj.170072, 29133539 PMC5687926

[9] Perry JJ Sivilotti MLA Émond M Stiell IG Stotts G Lee J . Prospective implementation of the Ottawa subarachnoid Hemorrhage rule and 6-hour computed tomography rule. Stroke. (2020) 51:424–30. doi: 10.1161/STROKEAHA.119.026969, 31805846

[10] Godwin SA Cherkas DS Panagos PD Shih RD Byyny R Wolf SJ . Clinical policy: critical issues in the evaluation and management of adult patients presenting to the emergency department with acute headache. Ann Emerg Med. (2019) 74:e41–74. doi: 10.1016/j.annemergmed.2019.07.009, 31543134

[11] Edlow JA Fisher J. Diagnosis of subarachnoid hemorrhage: time to change the guidelines? Stroke. (2012) 43:2031–2. doi: 10.1161/STROKEAHA.112.664011, 22821612

[12] Hoh BL Ko NU Amin-Hanjani S Chou SH-Y Cruz-Flores S Dangayach NS . 2023 guideline for the management of patients with aneurysmal subarachnoid hemorrhage: a guideline from the American Heart Association/American Stroke Association. Stroke. (2023) 54:e314–70. doi: 10.1161/STR.0000000000000436, 37212182

[13] Perry JJ Alyahya B Sivilotti MLA Bullard MJ Emond M Sutherland J . Differentiation between traumatic tap and aneurysmal subarachnoid hemorrhage: prospective cohort study. BMJ. (2015) 350:h568. doi: 10.1136/bmj.h568, 25694274 PMC4353280

[14] Rinkel GJE Wijdicks EFM Hasan D Kienstra GEM Franke CL Hageman LM . Outcome in patients with subarachnoid haemorrhage and negative angiography according to pattern of haemorrhage on computed tomography. Lancet. (1991) 338:964–8. doi: 10.1016/0140-6736(91)91836-J, 1681340

[15] van Gijn J van Dongen KJ Vermeulen M Hijdra A. Perimesencephalic hemorrhage: a nonaneurysmal and benign form of subarachnoid hemorrhage. Neurology. (1985) 35:493–7. doi: 10.1212/WNL.35.4.493, 3982634

[16] Westerlaan HE van Dijk JMC Jansen-van der Weide MC de Groot JC Groen RJM Mooij JJA . Intracranial aneurysms in patients with subarachnoid hemorrhage: CT angiography as a primary examination tool for diagnosis — systematic review and meta-analysis. Radiology. (2011) 258:134–45. doi: 10.1148/radiol.10092373, 20935079

[17] Menke J Larsen J Kallenberg K. Diagnosing cerebral aneurysms by computed tomographic angiography: meta-analysis. Ann Neurol. (2011) 69:646–54. doi: 10.1002/ana.22270, 21391230

[18] White PM Wardlaw JM. The non-invasive detection of intracranial aneurysms: are neuroradiologists any closer to replacing catheter angiography? Lancet Neurol. (2003) 2:444–55. doi: 10.1016/S1474-4422(03)00432-1

[19] Vlak MHM Algra A Brandenburg R Rinkel GJE. Prevalence of unruptured intracranial aneurysms, with emphasis on sex, age, comorbidity, country, and time period: a systematic review and meta-analysis. Lancet Neurol. (2011) 10:626–36. doi: 10.1016/S1474-4422(11)70109-0, 21641282

[20] Wermer MJH van der Schaaf IC Algra A Rinkel GJE. Risk of rupture of unruptured intracranial aneurysms in relation to patient and aneurysm characteristics. Stroke. (2007) 38:1404–10. doi: 10.1161/01.STR.0000260955.51401.cd, 17332442

[21] Alons IME Goudsmit BFJ Jellema K van Walderveen MAA Wermer MJH Algra A. Yield of computed tomography (CT) angiography in patients with acute headache, normal neurological examination, and normal non contrast CT: a meta-analysis. J Stroke Cerebrovasc Dis. (2018) 27:1077–84. doi: 10.1016/j.jstrokecerebrovasdis.2017.11.016, 29277281

[22] Edjlali M Gentric JC Régent-Rodriguez C . Does aneurysmal wall enhancement on vessel wall MRI help distinguish unstable intracranial aneurysms? Stroke. (2014) 45:3704–6. doi: 10.1161/STROKEAHA.114.006626 25325912

[23] Matouk CC Mandell DM Günel M Bulsara KR Malhotra A Hebert R . Vessel wall magnetic resonance imaging identifies the site of rupture in patients with multiple intracranial aneurysms: proof of principle. Neurosurgery. (2013) 72:492–6. doi: 10.1227/NEU.0b013e31827d1012, 23151622

[24] Mandell DM Mossa-Basha M Qiao Y Hess CP Hui F Matouk C . Intracranial vessel wall MRI: principles and expert consensus recommendations of the American Society of Neuroradiology. Am J Neuroradiol. (2017) 38:218–29. doi: 10.3174/ajnr.A4893, 27469212 PMC7963837

[25] Mossa-Basha M Alexander M Gaddikeri S . Vessel wall imaging for intracranial vascular disease evaluation. J Magn Reson Imaging. (2019) 50:1590–605. doi: 10.1002/jmri.26723 PMC5484417 26769729

[26] Nagahata S Nagahata M Obara M Kondo R Minagawa N Sato S . Wall enhancement of the intracranial aneurysms revealed by magnetic resonance vessel wall imaging using three-dimensional turbo spin-echo sequence with motion-sensitized driven-equilibrium: a sign of ruptured aneurysm? Clin Neuroradiol. (2016) 26:277–83. doi: 10.1007/s00062-014-0353-z, 25332151

[27] Carpenter CR Hussain AM Ward MJ Zipfel GJ Fowler S Pines JM . Spontaneous subarachnoid hemorrhage: a systematic review and meta-analysis describing the diagnostic accuracy of history, physical examination, imaging, and lumbar puncture with an exploration of test thresholds. Acad Emerg Med. (2016) 23:963–1003. doi: 10.1111/acem.12984, 27306497 PMC5018921

[28] Perry JJ Spacek A Forbes M Wells GA Mortensen M Symington C . Is the combination of negative computed tomography result and negative lumbar puncture result sufficient to rule out subarachnoid hemorrhage? Ann Emerg Med. (2008) 51:707–13. doi: 10.1016/j.annemergmed.2007.10.025, 18191293

[29] Bezov D Lipton RB Ashina S. Post-dural puncture headache: part I diagnosis, epidemiology, etiology, and pathophysiology. Headache. (2010) 50:1144–52. doi: 10.1111/j.1526-4610.2010.01699.x, 20533959

[30] Migdal VL Wu WK Long D McNaughton CD Ward MJ Self WH. Risk-benefit analysis of lumbar puncture to evaluate for nontraumatic subarachnoid hemorrhage in adult ED patients. Am J Emerg Med. (2015) 33:1597–601. doi: 10.1016/j.ajem.2015.06.048, 26189054 PMC4628593

[31] Greving JP Wermer MJH Brown RD Jr . Development of the PHASES score for prediction of risk of rupture of intracranial aneurysms. Lancet Neurol. (2014) 13:59–66. doi: 10.1016/S1474-4422(13)70263-1 24290159

[32] McDonald JS McDonald RJ Comin J . Risk of intravenous contrast material–mediated acute kidney injury: a propensity score–matched study stratified by baseline-estimated glomerular filtration rate. Radiology. (2014) 271:65–73. doi: 10.1148/radiol.13130775 24475854

[33] Chen J Zheng J Zhang Q Zhang J Dai Q Zhang D. Radiation exposure in recurrent medical imaging: identifying drivers and high-risk populations. Front Public Health. (2025) 13:1626906. doi: 10.3389/fpubh.2025.1626906, 40756407 PMC12315917

